# The impact of routine surveillance screening with magnetic resonance imaging (MRI) to detect tumour recurrence in children with central nervous system (CNS) tumours: protocol for a systematic review and meta-analysis

**DOI:** 10.1186/s13643-016-0318-1

**Published:** 2016-08-31

**Authors:** Caroline Main, Simon P. Stevens, Simon Bailey, Robert Phillips, Barry Pizer, Keith Wheatley, Pamela R. Kearns, Martin English, Sophie Wilne, Jayne S. Wilson

**Affiliations:** 1 Cancer Research UK Clinical Trials Unit (CRCTU), Institute of Cancer and Genomic Sciences, University of Birmingham, Birmingham, UK; 2 Sir James Spence Institute of Child Health, Royal Victoria Infirmary, Newcastle-Upon-Tyne, UK; 3 Centre for Reviews and Dissemination (CRD), University of York, York, UK; 4 Alder Hey Children’s NHS Foundation Trust, Liverpool, UK; 5 Birmingham Children’s Hospital NHS Foundation Trust, Birmingham, UK; 6 Queen’s Medical Centre, Nottingham University Hospitals’ NHS Trust, Nottingham, UK

**Keywords:** Children, Central nervous system tumours, Surveillance, Recurrent disease, Magnetic resonance imaging (MRI), Systematic review

## Abstract

**Background:**

The aim of this study is to assess the impact of routine MRI surveillance to detect tumour recurrence in children with no new neurological signs or symptoms compared with alternative follow-up practices, including periodic clinical and physical examinations and the use of non-routine imaging upon presentation with disease signs or symptoms.

**Methods:**

Standard systematic review methods aimed at minimising bias will be employed for study identification, selection and data extraction. Ten electronic databases have been searched, and further citation searching and reference checking will be employed. Randomised and non-randomised controlled trials assessing the impact of routine surveillance MRI to detect tumour recurrence in children with no new neurological signs or symptoms compared to alternative follow-up schedules including imaging upon presentation with disease signs or symptoms will be included.

The primary outcome is time to change in therapeutic intervention. Secondary outcomes include overall survival, surrogate survival outcomes, response rates, diagnostic yield per set of images, adverse events, quality of survival and validated measures of family psychological functioning and anxiety. Two reviewers will independently screen and select studies for inclusion. Quality assessment will be undertaken using the Cochrane Collaboration’s tools for assessing risk of bias. Where possible, data will be summarised using combined estimates of effect for time to treatment change, survival outcomes and response rates using assumption-free methods. Further sub-group analyses and meta-regression models will be specified and undertaken to explore potential sources of heterogeneity between studies within each tumour type if necessary.

**Discussion:**

Assessment of the impact of surveillance imaging in children with CNS tumours is methodologically complex. The evidence base is likely to be heterogeneous in terms of imaging protocols, definitions of radiological response and diagnostic accuracy of tumour recurrence due to changes in imaging technology over time. Furthermore, the delineation of tumour recurrence from either pseudo-progression or radiation necrosis after radiotherapy is potentially problematic and linked to the timing of follow-up assessments. However, given the current routine practice of MRI surveillance in the follow-up of children with CNS tumours in the UK and the resource implications, it is important to evaluate the cost-benefit profile of this practice.

**Systematic review registration:**

PROSPERO CRD42016036802

**Electronic supplementary material:**

The online version of this article (doi:10.1186/s13643-016-0318-1) contains supplementary material, which is available to authorized users.

## Background

The use of magnetic resonance imaging (MRI) scans in the diagnosis and management of children with central nervous system (CNS) tumours is well established [1]. Surveillance scans complement periodic history taking and physical examination and are undertaken based on the assumption that a tumour can re-occur before symptoms appear and that detection and treatment of recurrent disease before the development of signs or symptoms may improve outcome. The scheduling and imaging techniques used for surveillance protocols are therefore loosely based on the biological characteristics of the different CNS tumour types, taking into account the rate of tumour growth, the location and patterns of local and metastatic recurrence [2, 3].

Magnetic resonance imaging is a complex modality, with different sequences (protocols) being used by different centres, with the potential for scan results to be indeterminate. Clinicians face the challenge of diagnosing and managing patients that have new or old lesions seen on follow-up MRI and determining whether the lesion is pseudo-progression, radiation necrosis or tumour recurrence. This is further complicated by differences over time and between centres in radiological response definitions, complexities of scan interpretation and scan reader inter-rater reliability.

Obviously, earlier detection and treatment of recurrence needs to bestow a clinical benefit in terms of reduction in mortality or an improvement in quality of survival [4] in order for early detection strategies to be worthwhile, and further treatment strategies need to be available to balance the risk-benefit profile of earlier detection. These risks include the need for sedation or anaesthesia in children, the negative psychological impact associated with an upcoming imaging session and the consequent results and, at a societal level, the direct and in-direct healthcare costs incurred with surveillance screening and changes in patient management strategies.

The impact of surveillance imaging has been assessed in children with different types of malignant CNS tumours including low- and high-grade gliomas [5–7], medulloblastoma [6, 8–10], ependymoma, [11] and central nervous system primitive neuroectodermal tumours (CNS-PNETs) [12]. However, differences in imaging schedules and modalities (including both computerised tomography (CT) and MRI), and a reliance upon case series studies, mean no consensus has been reached regarding the utility of surveillance imaging, the optimal interval for undertaking scans or the length of surveillance following diagnosis. Whilst appropriate assessment of the impact of routine MRI surveillance screening to detect tumour recurrence in children with either no new, stable or improved neurological signs or symptoms with malignant CNS tumours is methodologically challenging and needs to be founded on data from appropriately designed randomised controlled trials (RCTs), given its current use in the UK, it is important to assess the cost-benefit of this practice. To date, no systematic reviews have been conducted that evaluate the impact of this screening strategy compared to imaging upon presentation of signs or symptoms of recurrent disease. This review therefore aims to:

1. Assess the impact of routine MRI surveillance to detect tumour recurrence in children with either no new, stable or improved neurological signs or symptoms with CNS tumours compared to alternative follow-up practices, including periodic clinical history taking and physical examination and the use of non-routine imaging upon presentation with disease signs or symptoms
2. Where possible, evaluate the effects of varying MRI screening intervals by tumour type and determine the optimum length of time for screening post initial diagnosis
3. Identify gaps and methodological weaknesses in the current evidence base to inform the design and analysis of further RCTs and make recommendations on the need for further primary research

## Methods

Standard systematic review methodology aimed at minimising bias will be employed, and reporting will follow the Preferred Reporting Items for Systematic Reviews and Meta-Analyses (PRISMA) guidelines [13]. The protocol for this review is registered with PROSPERO (CRD42016036802), available from http://www.crd.york.ac.uk/PROSPERO/display_record.asp?ID=CRD42016036802. The PRISMA for Protocols (PRISMA-P) checklist for the review is also included as Additional file 1.

### Data sources and searches

This review forms part of a wider work programme of systematic reviews which aim to assess the effects of different interventions for the treatment of CNS tumours in children, adolescents and young adults. Searches have therefore been conducted for studies examining the effects of imaging, surgery, radiotherapy, chemotherapy, immunotherapy, hormone therapy and biological therapies used alone or as part of a multimodality treatment regimen for all types of paediatric brain tumours. No study design filters have been applied to the searches. Specific details of the searches conducted are detailed below.

#### Bibliographic databases

A comprehensive, broad search strategy was developed using a combination of medical subject headings (MeSH) and free-text terms. The searches were limited by date from 1985 to October week 4, 2015. No language or publication status restrictions were applied, and ongoing studies were included.

The searches for published studies were undertaken using the following databases: MEDLINE (OvidSP); MEDLINE In-Process Citations & Daily Update (OvidSP); EMBASE (OvidSP); Cochrane Database of Systematic Reviews (CDSR) (Wiley); Cochrane Central Register of Controlled Trials (CENTRAL) (Wiley); CINAHL Plus (EBSCO); DARE (CRD website); and HTA (CRD website). The search strategy used for the MEDLINE search is reported in Appendix 1.

Grey literature, completed and on-going studies were identified by searches of NIH Clinical Trials (http://www.clinicaltrials.gov/); Current Controlled Trials (http://www.controlled-trials.com/); and WHO International Clinical Trials Registry Platform (ICTRP) (http://www.who.int/ictrp/en/)

#### Other sources

Experts in the field, from both the Project Advisory and Patient and Public Involvement (PPI) Groups, were contacted with a list of identified studies to find out whether they had knowledge of any further studies that had not been retrieved by the electronic searches. Reference lists of all studies included in the present review will be checked and citation searching undertaken in order to identify any further studies not retrieved by the electronic searches.

All identified references have been downloaded into Endnote X7 software for initial assessment and handling. Where flexibility is needed throughout the work programme for reference management and handling, Endnote software will be linked to bespoke Access databases in order facilitate sorting and manipulation of data items within indexed fields and abstracts.

### Study selection

All studies have been loosely ‘tagged’ according to the study design and type of intervention using the seven intervention categories outlined above. All studies ‘tagged’ as ‘imaging protocols’ will be used to form the potential pool from which studies will be screened against the specific inclusion criteria. Study selection will be undertaken by two reviewers working independently initially using the titles/abstracts from the pool of potential studies. Studies marked for inclusion by either reviewer will then undergo full independent text assessment. Any discrepancies will be resolved by recourse to the abstracts or full texts or through consensus with a third reviewer. A PRISMA flow chart illustrating the study selection process will be documented [13].

### Inclusion/exclusion criteria

- *Population*: Infants, children and young adults (up to age 25 years) with diagnoses of any type of CNS tumour that has either no new, stable or improved neurological signs or symptoms at the time of study recruitment. These include but are not limited to high- and low-grade gliomas (HGG and LGG), medulloblastoma, ependymoma and germ cell tumours. Studies that include both children and adults within the relevant populations will be included provided results are reported separately for children (defined as up to the age of 25). Likewise, data from studies including children with different CNS tumour types will be included provided that (a) data are reported separately by tumour type and (b) data are reported for ten or more participants with a specific type of tumour.
- *Interventions*: Routine interval follow-up MRI scans in children with either no new, stable or improved neurological signs or symptoms. These can be conducted at any screening interval determined within the primary study and include T2, T1, T1 with contrast, diffusion and FLAIR images. Studies that do not report the use of a post-operative MRI scan or use CT as the imaging modality at baseline will be excluded. Likewise, studies that use both MRI and CT scans for routine imaging surveillance will only be included if data are reported separately for children who underwent MRI scans.
- *Comparator*: Any alternative follow-up schedule, including the use of periodic clinical history taking and physical examination and non-routine scheduled MRI scan(s) conducted due to physical signs/symptoms of tumour progression or recurrence.
- *Outcomes*: The primary outcome is time to change in therapeutic intervention. Secondary outcomes are overall survival, surrogate survival outcomes, response rates, short- and long-term adverse events, diagnostic yield per set of images, quality of survival and validated measures of family psychological functioning and anxiety.
- *Study designs*: Randomised controlled trials (RCTs) and non-randomised comparative studies. All uncontrolled study designs and diagnostic test accuracy studies will be excluded.

### Data extraction

Data will be recorded on a standard data extraction form developed in either Access or Excel. The data will be extracted by one reviewer and checked by a second for accuracy. Any discrepancies will be resolved by recourse to the paper. Data from studies with multiple publications will be extracted and reported as a single study.

Data will be extracted on general (study name, study group (if applicable), publication date(s), principal investigator/authors); eligibility and study participants (e.g. tumour type and location; grade; age, gender, prior treatment history); definition of radiographic recurrence and other outcomes; intervention and comparator: MRI sequencing schedule (including plane(s), weighting, contrast enhancement; number of scans; scanning intervals; diagnostic yield per set of images; concomitant therapy); treatment intent (curative or palliative), study design (randomised controlled trial or non-randomised comparative study), length of follow-up and timing of outcome assessments; outcome measures (protocol specified, where available and reported); results (time to change in therapeutic intervention; overall survival, surrogate survival outcomes, response rates, short- and long-term adverse events, quality of survival and measures of family psychological functioning and anxiety); analysis methods (ITT or per protocol) and the author’s conclusions.

### Assessment of risk of bias in studies

The quality of RCTs and non-randomised comparative trials will be assessed using the Cochrane Collaboration’s risk of bias tool for randomised trials and ROBINS-I, respectively [14, 15].

Additional criteria will also be used to assess the adequacy of the sample size and methods of analyses and the likely external validity of the study. All assessment will be at the overall study level, not at the level of the individual outcomes. Quality assessment will be undertaken by one reviewer independently and checked for accuracy by a second. Any disagreements will be resolved by recourse to the study paper(s) and a third reviewer will be consulted where necessary. In addition to the methodological criteria listed above, the GRADE framework may be used to consider inconsistency between studies, precision of results, likelihood of publication bias and applicability of results to population(s) of interest [16].

### Data synthesis and analysis

#### Narrative synthesis

A narrative synthesis of study results will be presented (including text, figures and tables), to provide adequate interpretation of study findings. Studies will be grouped by tumour type, treatment line (induction, consolidation, salvage) and imaging sequences (where possible). The outcomes considered include time to change in therapeutic intervention, overall survival, surrogate survival outcomes, response rates, diagnostic ‘yield’ rate, quality of survival and validated measures of family psychological functioning and anxiety. Therefore, outcomes will be expressed in terms of hazard ratios (HR; (adjusted or unadjusted)), risk ratios (RR) and weighted mean differences as appropriate. All analyses will be conducted per outcome, including all studies that have reported data for the outcome.

Where more than one RCT has addressed the same question within the same tumour type and treatment line, and they are considered to be clinically similar (based on patient population and imaging protocol), results will be combined in a standard pairwise meta-analysis using assumption-free methods. All analyses will be carried out on an intention-to-treat (ITT) basis where possible, using the HR or RR as appropriate.

Heterogeneity will be investigated visually using forest plots and statistically using the *I*^2^ and *Q* statistic [17]. Heterogeneity will be formally investigated using sub-group analyses and meta-regression where sufficient data are available. It is anticipated that the effects of the following variables will be investigated: methodological quality of the primary studies; tumour type, location and grade; extent of resection; prior treatments (chemotherapy/radiotherapy/multimodal regimens); number of relapses; imaging protocols and imaging intervals. Other variables considered relevant on further examination of the literature or input from clinical experts may also be considered. The coefficient describing the effect of each variable on the outcome will be modelled, using a random effects model. All analyses will be conducted using RevMan (version 5.1) and STATA (STATA™ for Windows, version 10.1, Stata Corp; College Station, TX).

##### Assessment of small study effects

For each meta-analysis containing ten or more studies, the likelihood of small study effects and publication bias, namely the tendency for smaller studies to provide more positive findings, will be assessed using a modified linear regression test for funnel plot asymmetry as recommended where there are sufficient numbers of trials (i.e. six trials) [18].

## Discussion

The methodology used to conduct this review has been designed to be robust, comprehensive and minimise bias. However, it is anticipated there will be a number of limitations with the review. The assessment of the impact of routine MRI surveillance in children with malignant CNS tumours is complex and presents a number of methodological challenges. These relate to the natural history of the tumour types and highly different baseline risks of progression [19], differences in imaging technologies and definitions of radiological response between studies and changes in both of these over time, the type of imaging protocols and schedules selected and the complexity of scan interpretation and inter-rater reliability. Furthermore, the assessment and measurement of time from initial diagnosis to change in therapeutic intervention as the appropriate outcome is important, as measures of overall or surrogate survival outcomes will be confounded by changes in the treatment pathway due to potentially earlier radiological tumour recurrence detection. In terms of time to change in therapeutic intervention, the concepts of lead time and length bias are well documented in the screening literature [19], with lead-time bias minimised by measuring this outcome from initial diagnosis, not from the time of tumour recurrence [19]. Differences in time to change in therapeutic intervention due to recurrence associated with length bias; whereby longer time to change in therapeutic intervention may be due to inherent differences in the baseline tumour characteristics rather than due to earlier detection can be minimised through the use of adequately randomised, and therefore balanced, randomised controlled trials (RCTs) [19]. Whilst limiting the evidence base to controlled trials should strengthen the robustness of the evidence base, it may restrict the breath of the review where no trials for some tumour types or disease stages are available. This will impact the utility of the review results for informing decision-making on the wider effectiveness of surveillance MRI screening in children with CNS tumours. However, given the current routine practice of MRI surveillance in the follow-up of children with CNS tumours, it is important to evaluate the cost-benefit profile of this practice and the impact of different screening intervals in a methodologically robust manner.

## Dissemination

To ensure that our finding have clinical impact on patients, their parents and the physicians who care for them, results will be disseminated broadly by presenting at scientific conferences, published in peer-reviewed journals and through our Patient and Public Involvement (PPI) Partners who work for established high-profile UK brain tumour charities and our Clinical Steering Group.

## Appendix 1: clinical effectiveness search strategy

MEDLINE (OvidSP): 1985 - October week 4, 2015

1. Glioma/or Brain Neoplasms/or Meningioma/or Glioblastoma/or Astrocytoma/

2. ((brain or brainstem or intracranial or posterior fossa) adj3 (cancer* or carcinom* or tumor* or tumour* or neoplasm*)).mp.

3. (Astrocytoma* or Brain Stem Glioma* or Medulloblastoma*or Primitive Neuroectodermal Tumo?r* or ganglioneuroblastoma* or CNS neuroblastoma* or Ependymoblastoma or Medulloepithelioma or Pineal Parenchymal Tumour* or (Atypical Teratoid adj1 tumo?r*) or Oligoastrocytoma or ((Pilocytic or Gemistocytic) adj1 astrocytoma*) or ependymoma or primitive neuroectal tumo?r*).mp.

4. (((Diffuse fibrillary or Gemistocytic or Pilocytic Pilomyxoid Protoplasmic Subependymal giant cell) adj1 astrocytoma*) or Oligoastrocytoma or Oligodendroglioma or Oligoastrocytoma or Pleomorphic xanthoastrocytoma or ((astrocytoma or oligoastrocytoma or oligodendroglioma) adj1 astrocytoma*) or Glioblastoma or Gliomatosis cerebri or Gliosarcoma or ((diffuse intrinsic pontine glioma or low grade brain stem) adj1 glioma) or ((classic or desmoplastic or nodular or large cell or nodularity) adj1 medulloblastoma*) or Primitive Neuroectodermal Tumo?r* or ((ganglioneuroblastoma or neuroblastoma) adj 1central nervous system*) or Ependymoblastoma or Pineoblastoma or pineal parenchymal tumo?r* or (central nervous system adj1 atypical teratoid) or (central nervous system adj 1 rhabdoid tumo?r*) or Germinomas or ((immature or mature or malignant transformation) adj2 teratomas)).mp.

5. 1 or 2 or 3 or 4

6. exp Surgical Procedures, Operative/

7. surg*.mp.

8. debulk*.mp.

9. cytoreduc*.mp.

10. 6 or 7 or 8 or 9

11. (chemotherap* or antineoplastic agents or cytotoxic or alkylating agents or nitrosoureas or antimetabolite* or antitumor?r or ((antibod* or monoclonal) adj 3 Human*) or plant alkyloid* or (hormone* adj 1 agent*) or anthracycline* * or systemic therap*).mp.

12. (Everolimus or Afinitor or Cetuximab or Erbitux or Bevacizumab or Avastin or Cediranib or Recentin or lomustine or CCNU or CeeNU or carmustine or BiCNU or Carustine or Ethylnitrosourea or Streptozocin or Sorafenib or Nexavar or tipifarnib or Zarnestra or Erlotinib or Tarceva or Sorafenib or Nexavar or temsirolimus or Torisel or Sunitinib or Sutent or irinotecan or Camptosar or Campto or Vandetanib or Caprelsa or Cabozantinib or Cometriq or XL184 or Axitinib or AG013736 or Inlyta).mp.

13. 11 or 12

14. exp Immunotherapy/ae, cl, ct, mt, mo, nu, px, st

15. exp Genetic Therapy/ae, cl, ct, mt, mo, nu, ut

16. exp Imaging, Three-Dimensional/or exp Whole Body Imaging/or exp Magnetic Resonance Imaging/

17. exp Tomography, Emission-Computed/or exp Four-Dimensional Computed Tomography/or exp Tomography/or exp Tomography, Emission-Computed, Single-Photon/or exp Positron-Emission Tomography/

18. 16 or 17

19. (radiation therapy or radiotherap* or intensity modulat* radiotherapy*or radiosurgery or radiation oncology or reduced boost volume radiotherap* or hyper fractionat* stereotactic radiotherap*or adjuvant radiotherap* or body radiotherap* stereotactic*or computer assisted radiotherap*or computer assisted radiotherap*planning or conformal radiotherap* or dosage* radiotherap* or dose fractionation* radiotherap* or high energy radiotherap*or implant radiotherap*or intensity or modulated radiotherap*or interstitial radiotherap*orimage guided radiotherap*or stereotactic*guid* radiotherap* or local therap* or proton therap* or proton adj2 therap* or proton beam therap* or proton adj2 radiation or proton radiation therap* or proton adj2 radiotherap* or proton adj2 irradia* or PBT).mp.

20. 10 or 13 or 14 or 15 or 18 or 19

21. 5 and 20

22. (Response or overall survival or progression* free survival or event* free survival or time to recurrence or time to progression or disease* free interval* or endocrinopath* or ((growth or thyroid) adj 1 hormone adj 3 deficienc*) or ((glucocorticoid or gonadotropin) adj 3 deficienc*) or endocrine dysfuct* or (cardiac function* adj 3 impair*) or ataxia or spastic paresis or visual dysfunction or epilepsy or hemiparesis or neurolog* deficit*).mp.

23. 21 and 22

24. limit 23 to (yr = "1985 -Current" and ("newborn infant (birth to 1 month)" or "infant (1 to 23 months)" or "preschool child (2 to 5 years)" or "child (6 to 12 years)" or "adolescent (13 to 18 years)" or "young adult (19 to 24 years)") and humans)

## Additional file

Additional file 1: PRISMA-P completed checklist for protocol: the impact of routine surveillance screening with magnetic resonance imaging (MRI) to detect tumour recurrence in children with central nervous system (CNS) tumours. (DOCX 17 kb)

## Abbreviations

CNS: Central nervous system—the part of the nervous system consisting of the brain and the spinal cord
CT: Computerised tomography—radiography in which a three-dimensional image of a body structure is constructed by computer from a series of plane cross-sectional images made along an axis
HGG: High-grade glioma—high-grade gliomas encompass the WHO grade III gliomas (anaplastic astrocytoma) and grade IV gliomas (glioblastome multiforme)
LGG: Low-grade gliomas—tumours that exhibit glial differentiation and lack high-grade findings such as microvascular proliferation and necrosis
MRI: A technique that uses a magnetic field and radio waves to create detailed images of the organs and tissues within the body
